# Working hours indirectly affect anxiety symptoms through sleep and stress in dentists, physicians, and psychotherapists

**DOI:** 10.2478/aiht-2025-76-3954

**Published:** 2025-06-30

**Authors:** Patricia Tomac, Iva Japundžić Rapić, Liborija Lugović-Mihić, Jelena Macan, Adrijana Košćec Bjelajac

**Affiliations:** Institute for Medical Research and Occupational Health, Zagreb, Croatia; Bagatin Clinic, Zagreb, Croatia; University of Zagreb School of Dental Medicine, Zagreb, Croatia; Sestre Milosrdnice University Hospital Centre, Department of Dermatovenereology, Zagreb, Croatia

**Keywords:** Cohen’s Perceived Stress Scale, healthcare workers, helping professions, path analysis, sleep duration, sleep quality, working time, Zung Self-Rating Anxiety Scale, analiza puta, Cohenova ljestvica percipiranog stresa, kvaliteta spavanja, pomagačka zanimanja, radno vrijeme, trajanje spavanja, zdravstveni radnici, Zungova ljestvica za samoprocjenu anksioznosti

## Abstract

The aim of this study was to examine the effects of working hours on anxiety symptoms in highly educated helping professionals, especially through sleep duration, perceived stress and sleep quality. We analysed the results of 172 helping professionals (dentists, physicians, and psychotherapists, 57 % women, average age 25–63 years) who had participated in a larger study examining the effects of working conditions and constitutional factors on the onset of hand eczema. The participants answered a battery of questionnaires, including Cohen’s Perceived Stress Scale, Zung Self-Rating Anxiety Scale, and a set of standard questions on sociodemographic characteristics, working hours, sleep, and job characteristics. They also underwent clinical examination of skin on hands and wrists. Participants reported working between 19 and 90 h a week, with 25.1 % working more than 50 h a week. Several path analysis models used in the study showed that working hours predicted anxiety only indirectly. The final model “working hours → sleep duration on workdays → perceived stress → anxiety symptoms” showed excellent fit [χ^2^(14)=10.345; P>0.05; CFI=1.000; RMSEA 90 % CI (0.000, 0.054); P>0.05; SRMR=0.028]. Our results indicate that long working hours are associated with shorter sleep duration, which, in turn, is associated with higher levels of perceived stress and subsequently higher levels of anxiety. Long working hours in highly educated helping professionals may therefore put at risk their own health and safety and that of the recipients of their services.

## Abstract (hr)

Cilj ovog istraživanja bio je ispitati odnos između radnog vremena i simptoma anksioznosti kod visokoobrazovanih stručnjaka pomagačkih profesija, s obzirom na neizravne učinke radnog vremena kroz trajanje spavanja, percipirani stres i kvalitetu spavanja. Analizirali smo rezultate 172 stručnjaka pomagačkih profesija (stomatolozi, liječnici i psihoterapeuti; 57 % žena, 25 – 63 godine) koji su sudjelovali u širem projektu ispitivanja utjecaja radnih uvjeta i konstitucijskih čimbenika na nastanak ekcema šaka. Sudionici su odgovarali na skup upitnika, uključujući Cohenovu ljestvicu percipiranog stresa, Zungovu ljestvicu za samoprocjenu anksioznosti i skup standardnih pitanja o sociodemografskim karakteristikama, radnom vremenu, spavanju i karakteristikama posla. Također su bili podvrgnuti kliničkom pregledu kože na rukama i zapešćima. Sudionici su izjavili da tjedno rade između 19 i 90 sati, a njih 25,1 % radilo je više od 50 sati tjedno. Evaluirali smo nekoliko modela analize puta kako bismo ispitali izravne i neizravne učinke radnog vremena na simptome anksioznosti putem spavanja i stresa. Očekivano, radno vrijeme nije izravno predviđalo anksioznost. Konačni model „radno vrijeme → trajanje spavanja radnim danima → percipirani stres → simptomi anksioznosti“ pokazao je izvrsno podudaranje s podatcima [χ^2^(14)=10,345; P>0,05; CFI=1,000; RMSEA 90 % CI (0,000; 0,054); P>0,05; SRMR=0,028]. Naši rezultati pokazuju da je odnos između dugog radnog vremena i anksioznosti kod visokoobrazovanih stručnjaka pomagačkih profesija neizravan skraćivanjem trajanja glavnog spavanja, za koje je poznato da povećava emocionalnu reaktivnost na svakodnevne stresore. Dugo radno vrijeme visokoobrazovanih stručnjaka pomagačkih profesija stoga može predstavljati rizik za njihovo vlastito zdravlje i sigurnost te za zdravlje i sigurnost primatelja njihovih usluga.

Time spent at work has declined from the end of the 19^th^ to the end of 20^th^ century, but this decline has been uneven across countries and occupations (1). In lower- to middle-income countries workers work longer hours than in high-income countries (2). Since the COVID-19 pandemic, this gap has increased even further (3). In high income countries, some sectors, including professional, scientific, and technical services (3) have seen a decrease in total working hours.

A number of studies have shown that long working hours are associated with negative health outcomes like cardiovascular diseases, metabolic syndrome, mortality risk, and sleep disorders (4,5,6). One of the reasons that the long tradition of studying the relationship between working time and workers’ wellbeing has been revived is the increase in cognitive and emotional demands of work and constant availability of workers made possible by information technology (7). Working overtime entails prolonged exposure to work-related psychosocial stressors identified as leading risk factors for mental health impairment in workers (8,9,10). In turn, mental health issues – depression, anxiety, and stress in particular – are the leading causes of long sickness absenteeism (11,12,13). Long working hours also leave less time for recovery and important social activities (14,15,16,17). This work-life imbalance can affect the sleep-wake rhythm, and the evidence of poor sleep affecting workers’ physical and mental health is abundant (17,18,19,20). Unlike depression, anxiety has received less attention as a consequence of poor sleep, and its relationship with arousal and sleep is complex, but the literature suggests that poor sleep can be viewed as both the cause and consequence of anxiety (21, 22). Inadequate sleep duration and quality lead to poor recovery, impaired daytime functioning, sleepiness, fatigue, heightened emotional reactivity, and greater vulnerability to stress (20, 23, 24). Sleep deprivation is also associated with a mild and transient increase in stress-related neuroendocrine activity (25, 26), which obstructs sleep and rest and leads to at least transient sleep impairment (23, 27, 28). Since sleep loss and stress both contribute to the allostatic load, there are models that incorporate stress and sleep indicators to predict the related health outcomes (29, 30).

The aim of our study was to shed more light on the complex relationship between working hours and mental health of highly educated professionals by investigating such models. We started from the assumption that working hours were related to anxiety indirectly, through the effects on sleep and stress. Since sleep and stress may be reciprocally related, we wanted to examine which underlying mechanism was more likely; “stress → sleep” or “sleep → stress”. We hypothesised that working longer hours would negatively impact sleep duration and quality on workdays and that poor sleep would be associated with higher perceived stress, which would, in turn, increase anxiety. The alternative hypothesis was that working longer hours would shorten sleep on workdays, which would lead to poorer sleep quality and higher perceived stress. Increased perceived stress would also worsen sleep quality and would increase anxiety symptoms, both directly and indirectly, by affecting sleep quality.

## PARTICIPANTS AND METHODS

This study was a part of a larger cross-sectional field study that examined the impact of working conditions and constitutional factors on the onset of hand eczema in medical doctors and dental medical doctors in Zagreb, Croatia (31,32,33). In that study other highly educated professionals who worked in immediate contact with people but did not wear protective gloves served as controls. Recruitment details have been described elsewhere (31,32,33).

### Participants

This study included 172 workers (57 % women) in helping professions, namely dentists, physicians, and psychotherapists. From the original sample of 185 participants we excluded 11 who were not helping professionals and were not in direct contact with patients/clients and two participants who were older than 65 years at the time of the study, which is the legal age of retirement in Croatia.

We focused on highly educated professionals, as they run higher psychosocial risks due to the nature of their work, i.e., dealing with people in various forms of distress, and due to their long working hours.

### Sleep and job characteristics

The participants answered an extensive health questionnaire compiled for the purpose of the main study (31), which also comprised a set of standard questions on sleep and job characteristics, as well as standardised instruments for stress and anxiety assessment. The participants reported their sleep characteristics during the past month: bedtime (hh:mm), wake-up time (hh:mm), nap duration on workdays, free days, and total nap duration (hours and minutes), use of sleeping aids (0 – No, 1 – Yes), subjective estimation of sleep latency (minutes), subjective estimation of sleep quality (1 – Very bad to 5 – Excellent), and self-reported sleep need (hours and minutes). Sleep duration was calculated as difference between wake-up time and time of falling asleep (bedtime + sleep latency). Mid-sleep on free days was used as a measure of chronotype, calculated as proposed by Roenneberg et al. (34, 35).

Assessed job characteristics were: profession, level and type of postgraduate education, work experience (years), average weekly hours on the main job, engagement in additional paid job (0 – No, 1 – Yes), average weekly hours on additional job, shift work (0 – No, 1 – Yes), beginning and end of each shift in hours and minutes, number of days on call, number of free and vacation leave days during last month. Weekly working hours were calculated as the sum of hours spent at the main and additional job over a week.

### Perceived stress and anxiety

Participants answered the Cohen’s Perceived Stress Scale (PSS) (36) by rating 10 questions about their thoughts and feelings during the past month on a scale from 0 – Never to 4 – Very often. The total PSS score can range between 0 and 40. Scores greater than 20 indicate clinically significant levels of perceived stress. The participants also answered the Zung Self-Rating Anxiety Scale (SAS) (37), whose total score is the sum of scores on 20 questions about anxiety experienced over the past few days rated on a scale from 1 – A little of the time to 4 – Most of the time. The theoretical range is 20–80. Scores ≥36 indicate clinically significant levels of anxiety.

The participants also took clinical dermatological examination (31,32,33). In this study, we only report the information about percentage of participants presenting with symptoms of hand eczema as a confounding variable.

### Data analyses

The obtained data were analysed using the SPSS 27.0 (IBM Corp, Armonk, NY, USA) and Mplus Version 8.11 (38) statistical software. We used descriptive statistics to describe participants characteristics. Relationships between the variables in a model were analysed using correlation coefficients and scatterplot matrices and were found to be linear or approximately linear. Analyses of residual distributions indicated that the assumptions of normality and homoscedasticity were met in all regression models (assessed in the most complex indirect pathway: “working hours → sleep duration → perceived stress → sleep quality → anxiety symptoms”), except for the model examining the relationship between anxiety symptoms and subjective sleep quality.

The structural equation modelling (SEM) approach was used to examine the association between working hours and anxiety, both directly and indirectly (through sleep characteristics and perceived stress symptoms). For this purpose we developed three different path models using the same observed variables with varying structural paths. In all models, working hours served as the main independent variable, anxiety symptoms as the main dependent variable, while sleep duration, subjective sleep quality, and perceived stress symptoms were set as mediators between the two.

**Figure 1:**
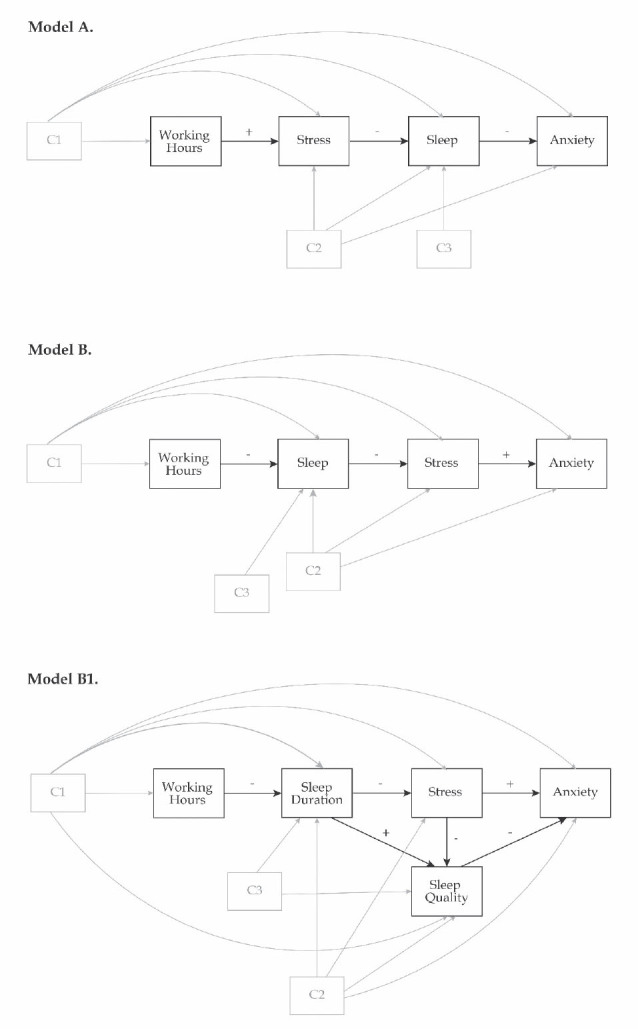
Three hypothesised alternative path models of the role of sleep and stress in the relationship between working hours and anxiety symptoms with potential confounders divided into three groups: C1) gender, profession, work experience (years), and shift work; C2) sleep duration on free days, mid-sleep time point, using sleep aid, and presence of hand eczema symptoms; and C3) sleep need and total weekly nap duration

We first defined Model 0 to evaluate the direct association between working hours and anxiety, as well as the indirect associations through each of the hypothesised mediators separately. This model included parallel mediators with their residuals allowed to covary. Next, we defined two alternative models (A and B, Figure 1) to analyse two different paths. In both models, sleep duration and sleep quality were specified as parallel mediators, with covarying residuals. The key distinction between these models is in the sequencing of the mediators: Model A assumed the “stress → sleep” path, whereas Model B assumed the “sleep → stress” path. Since Model B demonstrated a better fit to the data and greater theoretical merit, it was further modified to evaluate a more refined (Model B1) and more parsimonious (Model B2) solution. In Model B1, the direct path “working hours → sleep quality” was omitted, and a new direct path “sleep quality → anxiety symptoms” was added. Also, the covariation path between sleep duration and sleep quality was redefined to be directional path (“sleep duration → sleep quality”), and the original path “sleep quality → perceived stress” was modified to “perceived stress → sleep quality.” In Model B2, sleep quality was removed from the mediators’ list and included as a covariate instead.

All variables in the models were treated as continuous. To circumvent potential violations of normality, the analyses were conducted on bootstrapped samples (1000 times). Model parameters were estimated using maximum likelihood (ML) estimation. The statistical significance of the direct and indirect effects was evaluated based on the 95 % bootstrap confidence intervals (CI), and an effect was considered significant if the CI for a given estimate did not include zero. We used several indicators to evaluate the fit of the proposed models to the data. The chi-squared (χ^2^) test was used to evaluate overall model fit, the comparative fit index (CFI) and Tucker Lewis index (TLI) to evaluate incremental fit, and the root mean square error of approximation (RMSEA) and standardised root mean square residual (SRMR) as absolute fit indices. A non-significant χ^2^-test indicates that the model is consistent with the data, while a non-significant RMSEA supports the close-fit hypothesis (39). RMSEA values of 0.05 or less indicate close fit, whereas values greater than 0.10 suggest substantial error in approximation (40). To evaluate model quality, we adopted the following cut-off criteria: RMSEA≤0.06, SRMR<0.08, and CFI/TLI≥0.95 (41). To compare and rank the proposed models, we used the Akaike information criterion (AIC), where lower values indicate a higher relative quality of the model (42). We also used the sample-size-adjusted Bayesian Information Criterion (BIC).

In all models we controlled for the potentially confounding effects of gender, profession, work experience (years), working in shifts, sleep need, sleep duration on free days, total weekly nap duration, mid-sleep time point, using sleep aid, and the presence of hand eczema (Figure 1). More precisely, sleep variables were regressed on all ten confounders; anxiety and stress were regressed on gender, profession, work experience, working in shifts, sleep duration on free days, mid-sleep time point, using sleep aid, and the presence of hand eczema; and working hours were regressed on gender, profession, work experience, and working in shifts.

Finally, we conducted sensitivity analyses to evaluate the robustness of the fit across all proposed models when the effects of potential confounders were not controlled for.

Regarding the missing data treatment, we used listwise deletion, which we assumed appropriate, given that less than 5 % of values were missing (43): one participant did not have information for working hours and three participants had missing data for the outcome variable.

### Ethical considerations

The study was conducted in accordance with the Declaration of Helsinki, and the participants signed the informed consent form. The study was approved by the Ethics Committees of the Institute for Medical Research and Occupational Health, Zagreb (100-21/17-6), Sestre milosrdnice University Hospital Centre, Zagreb (EP-15006/17-3), University of Zagreb School of Dental Medicine, Zagreb (05-PA-26-3/2018), University Hospital Dubrava, Zagreb (3709-1/18), and Dental Outpatient Clinic, Zagreb (100-01/19-01). Participation was voluntary and anonymous, and the data were confidential.

## RESULTS

### Descriptive statistics and correlation findings

The total sample of participants included 74 dentists (43 %), 72 physicians (41.9 %), and 26 psychotherapists (15.1 %). Their mean age was 41.30±9.8 years. Apart from five dentists, all other participants completed postgraduate education (97 %). Participants’ work experience ranged from 0.5 to 39 (5.06 ±9.4) years and they reported working from 19 to 90 (45.41±12.59) h per week. 25.1 % reported working more than 50 h per week, and 28.5 % reported working in shifts (morning and afternoon). 28.5 % reported having additional highly skilled job (e.g., private practice in the same profession, teaching, consultant positions). 45 % (n=77) presented with at least one symptom of hand eczema at clinical examination.

Table 1 shows self-reported sleep parameters over the previous month. On workdays, participants slept significantly less than on free days, they went to bed and fell asleep earlier, and their total nap time was shorter.

**Table 1:** Descriptive parameters for sleep timings (hh:mm) and durations (decimal hours) on workdays, free days and across week in our sample of helping workers (N=172)

	**Workdays**				**Free days**				
	**M**	**SD**	**Min**	**Max**	**M**	**SD**	**Min**	**Max**	**P**
Bedtime	23:19	00:51	21:00	01:30	23:52	01:10	21:00	05:00	<0.001
Sleep onset	23:34	00:54	21:01	01:45	24:08	01:13	21:15	05:15	<0.001
Wake-up time	06:16	00:43	03:00	08:00	7:57	01:19	4:00	13:00	<0.001
Duration of main sleep	6.70	1.00	3.00	9.50	7.83	1.20	3.98	11.50	<0.001
Nap duration^#^	0.23	0.43	0.00	2.00	0.39	0.64	0.00	3.00	<0.001
**Across week / past month**						**M**	**SD**	**Min**	**Max**
Duration of main sleep (5*workdays, 2*free days)						7.02	0.90	3.71	9.50
Subjective sleep quality (1=very bad; 5=excellent)						3.68	0.90	1	5
Total nap duration^#^						0.62	0.90	0.00	4.25
Estimated sleep need						7.27	0.80	5.00	10.00
Mid-sleep on free days corrected for sleep debt						3:37	1:02	24:20	8:35

# Median=0

Ten participants reported having taken sleeping aid over the previous month: alcohol (one participant), chamomile tea (one participant), and benzodiazepines (eight participants).

On average anxiety symptoms were not significantly pronounced (30.78±6.114), and clinically significant levels were reported by 19 % of participants. Mean levels of perceived stress were also not elevated (15.01±5.764) and were clinically significant in 21 % of participants.

Table 2 shows the relationships between variables included in path models and full correlation matrix. Absolute values of significant correlations spanned from 0.150 to 0.619, and the correlation between perceived stress and anxiety symptoms was the highest.

**Table 2:** Correlation coefficients for all variables in path analysis models examining the relationship between working hours and anxiety (N=172)

	**AS**	**WH**	**SD_w_**	**SSQ**	**PSS**	**Gender**	**PR**	**WE**	**SWork**	**SNeed**	**SD_f_**	**Nap**	**SAid**	**MSFsc**
**WH**	−0.090													
**SD_w_**	−0.154^*^	−0.229^**^												
**SSQ**	−0.434^***^	−0.056	0.239^**^											
**PSS**	0.619^***^	−0.071	−0.238^**^	−0.353^***^										
**Gender (1=male, 2=female)**	0.179^*^	−0.193^*^	0.201^**^	0.044	0.177^*^									
**PR (0=dentists/doctors, 1=psychotherapists)**	−0.135	−0.105	0.295^***^	0.042	−0.182^*^	0.137								
**WE (years)**	−0.120	−0.129	−0.030	0.010	−0.165^*^	−0.047	0.243^**^							
**SWork (0=no, 1=yes)**	−0.018	−0.136	0.178^*^	0.138	−0.048	0.132	0.165^*^	−0.016						
**SNeed (decimal hours)**	0.051	−0.104	0.322^***^	0.041	0.010	0.256^***^	0.121	−0.108	0.141					
**SD_f_ (decimal hours)**	−0.050	−0.036	0.447^***^	0.127	−0.018	0.257^***^	0.089	−0.124	0.058	0.438^***^				
**Nap (decimal hours)**	0.089	0.051	−0.256^***^	−0.033	0.060	−0.268^***^	−0.217^**^	−0.036	−0.148	−0.153^*^	−0.174^*^			
**SAid (0=no, 1=yes)**	0.223^**^	−0.018	−0.109	−0.271^***^	0.216^**^	−0.035	0.103	0.078	−0.047	−0.005	−0.178^*^	−0.053		
**MSFsc**	−0.047	0.013	−0.150^*^	−0.002	−0.013	−0.086	0.047	−0.162^*^	0.029	0.026	−0.125	0.098	0.138	
**HE (0=no, 1=yes)**	−0.080	0.096	−0.059	0.008	0.033	−0.186^*^	−0.119	−0.020	0.209^**^	−0.086	−0.202^**^	0.005	0.026	0.013

* P<0.05;

** P<0.01;

*** P<0.001.

AS – anxiety symptoms; HE – hand eczema; MSFsc – mid-sleep on free days corrected for sleep debt; Nap – total weekly nap time; PR – profession; PSS – perceived stress scale; SAid – sleeping aids; SD_f_ – sleep duration on free days; SD_w_ – sleep duration on workdays; SNeed – sleep need; SSQ – subjective sleep quality; SWork – shift work; WE – work experience; WH – working hours

### Path analysis findings

Table 3 shows the global fit statistics for all five tested path models. Model 0, which specified parallel mediators, was fully saturated in terms of the paths between the independent, dependent, and mediating variables but retained 10 degrees of freedom, because not all variables were regressed on each confounding variable. As a result, Model 0 showed an excellent fit. In contrast, Model A, which assumed the indirect pathway “stress → sleep,” exhibited poor fit across all global fit statistics, with the exception of SRMR, which alone suggested an acceptable fit. Reversing the order of the mediators to “sleep → stress” (Model B) notably improved the model’s fit to the data. To further improve the fit, two revised versions of the Model B (B1 and B2) were developed as described in the Methods section. As shown in Table 3, both models demonstrated excellent fit.

**Table 3:** Fit statistics of tested models of the (indirect) relationship between working hours and anxiety symptoms

**Model**	**χ**	**P_χ^2^_**	**CFI**	**TLI**	**RMSEA (90% CI)**	**P_RMSEA_**	**SRMR**	**AIC**	**BIC^$^**
0: parallel mediators	6.331 (10)	0.7867	1.000	1.000	0.000 (0.000, 0.055)	0.935	0.022	4315.777	4314.638
A: stress → sleep	66.160 (14)	<0.001	0.765	0.000	0.147 (0.113, 0.184)	<0.001	0.054	4367.606	4366.542
B: sleep → stress	24.445 (14)	0.0405	0.953	0.798	0.066 (0.014, 0.108)	0.246	0.036	4325.891	4324.827
B1: sleep → stress → sleep	9.956 (14)	0.7654	1.000	1.000	0.000 (0.000, 0.052)	0.944	0.027	4311.402	4310.338
B2: SD_w_ → PSS	10.345 (14)	0.7366	1.000	1.000	0.000 (0.000, 0.054)	0.934	0.028	3853.865	3853.029

PSS – perceived stress scale; SD_w_ – sleep duration on workdays; $ – sample-size adjusted BIC (n*=(n+2)/24). Note: In all models the effects of potential confounders were controlled for as described in the Methods section. In Model B2, sleep quality was added to the confounders’ list for mediators and the outcome

### Direct and indirect effects

Next, we examined the direct and indirect effects in models that demonstrated good fit to our data. In all models we statistically controlled for the effects of potential confounders as described in the Methods section.

**Figure 2:**
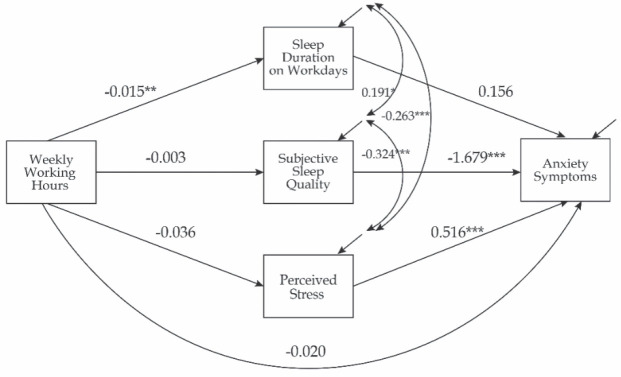
Results of the path analysis for model with parallel mediators and direct effect (Model 0; N=168). The confounding effects of gender, profession, work experience, shift work, sleep need, sleep duration on free days, total weekly nap duration, mid-sleep time point, using sleep aid, and presence of hand eczema symptoms (not shown) were controlled for as described in Methods section. Unstandardised coefficients and significances are shown (*P<0.05; **P<0.01; ***P<0.001)

In Model 0, the results confirmed no direct effect of working hours on anxiety symptoms (Figure 2) [β=−0.042, SE=0.066, bootstrap 95 % CI (−0.172, 0.088)]. Additionally, none of the tested indirect paths through the individual mediators were statistically significant: sleep duration on workdays [β=−0.005, SE=0.014, bootstrap 95 % CI (−0.032, 0.022)]; sleep quality [β=0.010, SE=0.020, bootstrap 95 % CI (−0.030, 0.050)]; or perceived stress [β=−0.038, SE=0.039, bootstrap 95 % CI (−0.116, 0.039)].

However, the analysis of the individual paths in the model revealed that longer working hours were associated with shorter sleep on workdays. Shorter sleep duration on workdays was associated with both poorer sleep quality and higher levels of perceived stress. Poor sleep quality was associated with higher levels of perceived stress. Both poor sleep quality and higher levels of perceived stress were significantly associated with higher levels of anxiety symptoms (Figure 2).

**Figure 3:**
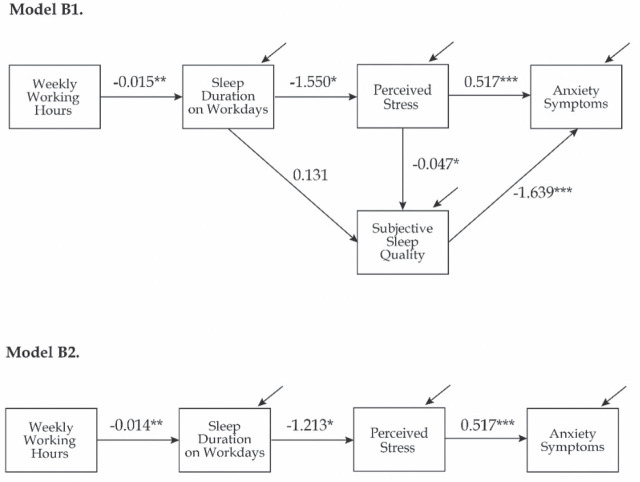
Results of the path analyses for the two models with the best fit (Models B1 and B2; N=168). The confounding effects of gender, profession, work experience, shift work, sleep need, sleep duration on free days, total weekly nap duration, mid-sleep time point, using sleep aid, and presence of hand eczema symptoms (not shown) were controlled for as described in Methods section. Unstandardised coefficients and significances are shown (*P<0.05; **P<0.01; ***P<0.001)

Model B1 tested three indirect pathways between working hours and anxiety symptoms, involving two or all three mediating variables in the following sequence: 1) “sleep duration → perceived stress,” 2) “sleep duration → sleep quality,” and 3) “sleep duration → perceived stress → sleep quality” (Figure 1, Model B1). All estimated path coefficients for Model B1 and B2 are presented in Table 4 and Figure 3. Among the tested pathways, two indirect pathways were statistically significant; “sleep duration → perceived stress” and “sleep duration → perceived stress → sleep quality” (bootstrap 95 % CI=[0.001, 0.009]). Interestingly, the direct effect “sleep duration → sleep quality” was not significant when perceived stress was in the equation.

**Table 4:** Direct, indirect, and total effects in the two models with the best fit (N=168)

**Model and effects**	**β**	**S.E.**	**P-value**	**Bootstrap 95% CI**
Model B1: sleep→stress→sleep				
*Direct effects*				
WH→SD_w_	−0.186	0.070	0.007	[−0.321, −0.045]
SD_w_→SSQ	0.145	0.098	0.138	[−0.047, 0.325]
PSS→SSQ	−0.298	0.072	<0.001	[−0.444, −0.159]
SD_w_→PSS	−0.270	0.074	<0.001	[−0.418, −0.133]
PSS→AS	0.488	0.074	<0.001	[0.332, 0.630]
SSQ→AS	−0.243	0.063	<0.001	[−0.373, −0.123]
*Indirect effects*				
WH→SD_w_→SSQ→AS	0.007	0.005	0.214	[−0.002, 0.018]
WH→SD_w_→PSS→AS	0.025	0.011	0.024	[0.006, 0.048]
WH→SD_w_→PSS→SSQ→AS	0.004	0.002	0.103	[0.001, 0.009]
*Total indirect effects*	0.035	0.014	0.015	[0.008, 0.065]
Model B2: sleep duration→stress				
*Direct effects*				
WH→SD_w_	−0.180	0.069	0.009	[−0.313, −0.047]
SD_w_→PSS	−0.211	0.072	0.003	[−0.352, −0.072]
PSS→AS	0.489	0.074	<0.001	[0.332, 0.630]
*Indirect (total indirect) effect*				
WH→SD_w_→PSS→AS	0.019	0.009	0.047	[0.004, 0.040]

AS – anxiety symptoms; CI – confidence interval; PSS – perceived stress scale; SD_w_ – sleep duration on workdays; SE – standard error; SQ – subjective sleep quality; WH – working hours. Note: In both models the effects of potential confounders were controlled for as described in the Methods section. Additionally, in Model B2, sleep quality was added to the confounder list for SD_w_, PSS, and the outcome

Model B2 had a single indirect pathway: “working hours → sleep duration → perceived stress → anxiety symptoms,” with subjective sleep quality included as a covariate. As expected, each path and the overall indirect effect were statistically significant (Table 4). As Model B2 had a lower AIC value, it was therefore identified as the preferred model (Table 3).

### Results of the sensitivity analysis

The evaluated models included a relatively large number of confounders, which increased model complexity. One disadvantage of adding many parameters is that it can lead to deflation of the chi-squared statistic (44) and produce more favourable fit indices that are derived from chi-squared, such as RMSEA, CFI, and TLI. Additionally, SRMR values may be reduced due to overfitting the observed covariance structure (45). In models B1 and B2, RMSEA, CFI, and TLI indicated perfect fit, most likely due to the chi-squared value being smaller than the degrees of freedom (46). To assess the robustness of the tested models, we performed additional path analyses for Models A, B, B1, and B2 without accounting for the effects of potential confounders. The fit indices for all models showed a slight decline, but both preferred models, B1 and B2, retained excellent fit. Specifically, Model B1 achieved the following fit indices: χ^2^(4)=4.701; P=0.319; CFI=0.995; TLI=0.987; RMSEA 90 % CI=0.032 (0.000, 0.124); P=0.522; SRMR=0.043; and AIC=2988.268. Similarly, Model B2 retained strong fit with: χ^2^(3)=3.647; P=0.302; CFI=0.993; TLI=0.987; RMSEA 90 % CI=0.036 (0.000, 0.138); P=0.477; SRMR=0.050; and AIC =2570.686. The significance of the individual paths remained unchanged, further supporting the robustness of findings.

## DISCUSSION

This study has shown that sleep and stress mediate the relationship between working hours and anxiety. More specifically, longer working hours are associated with shorter sleep duration on workdays, which is associated with higher perceived stress. Stress, in turn, is associated with higher anxiety. There is no evidence that working hours affect anxiety levels independently of their impact on sleep and stress.

Workers in helping professions are exposed to higher risk of stress and burnout due to different factors in their work environment, including high levels of responsibility and emotional strain (47). Highly skilled professionals tend to work long hours and take additional paid jobs within area of their expertise such as teaching, private practice, and/or consultancy, which was also observed in our study. Both caring/giving nature of their work and long working hours pose a psychosocial risk for their health and wellbeing. In our study, 52 % of participants worked overtime, of whom over 25 % exceeded the national legal limit of 50 h a week. Other studies show that working more than 55 h a week poses a significant risk for workers’ health and safety by increasing the risk of incidents (48), triggering sleep problems (49), such as sleep deprivation and increased fatigue and sleepiness (14), and by eliciting the development of depressive and anxiety symptoms, especially in women (50).

People differ in the degree to which they respond to stress with various non-specific sleep difficulties (24). Those whose sleep is more sensitive to stress are at a higher risk of developing insomnia. Sleep sensitivity to stress also seems to moderate the effect that stress-induced cognitive intrusions have on insomnia, amplifying the effect of the intrusions (51). In this context, it is important to point out that people suffering from insomnia are more likely to suffer from depression and anxiety than those without insomnia (52). Chronically disturbed sleep can contribute to the development of anxiety disorder by interfering with psychophysiological processes, i.e., allostatic load (22). On the other hand, optimal sleep duration (7–9 h a day) seems to protect against anxiety (53).

In our study longer working hours predict shorter duration of the main sleep during the work week. Our participants slept around an hour less than needed, and compensated for the lost sleep by napping over the work week and/or extending their sleep on days off (for 84 min on average). The duration of main sleep on workdays and self-reported sleep quality in our study are negatively associated with stress and anxiety symptoms. Kalmbach et al. (21) reported that the relationship between sleep duration and anxiety was bidirectional, but also that the effect of sleep duration on the development of anxiety was greater than the other way around. In that study, trainees who had significant sleep problems even before their internship were at a higher risk of cutting sleep and developing anxiety due to increased stress. The high correlation between perceived stress and anxiety symptoms in our study as well as taking benzodiazepine for sleep indicate impaired mental health in roughly 20 % of our participants. Our analyses show that this imbalance could partly be attributed to sleep deprivation as a consequence of long working hours. Impaired mental health in such professionals may pose a risk for the wellbeing of the very patients/clients that they are treating.

The relationship between working time and sleep, between sleep and stress, between stress, working time, and physical and mental health have already been studied. However, the exact mechanism of the negative impact of working hours on mental health outcomes is still not fully understood. What our study has shown is that working hours have no direct impact on either anxiety, perceived stress, or sleep quality, but only on sleep duration on workdays. This finding is expected, since people who do not work may also suffer from anxiety, just like long working hours may interfere with the fulfilment of other biological and social needs that we did not explore in this study. Similar to previous studies (9, 14, 21, 49, 54), we have found that working hours advance bedtime, sleep onset, and especially the wake-up time, which results in shorter sleep duration. The observed shortening of sleep could lead to inadequate psychophysiological recovery on workdays and act as a stressor increasing physiological, cognitive, and emotional reactivity (17, 23, 25). Being tense, reactive, tired, and having various physical symptoms, including headaches and gastrointestinal symptoms, are all states of anxiety and distress owed to sleep deprivation (22). Our study is consistent with other studies but also complements them with new knowledge on the mechanism of impact of working hours on anxiety via sleep and stress, which has been rarely examined in combination (29, 30).

The main advantage of the presented models is simultaneous analysis of these interrelated factors in a theoretically plausible causal order, while accounting for relevant confounders. However, the inclusion of numerous confounders increased model complexity and likely contributed to the deflation of chi-squared values, which in models B1 and B2 fell below the degrees of freedom. Such conditions can lead fit indices to overstate model adequacy, as reflected in the perfect fit indicated by RMSEA, CFI, and TLI in B models. Therefore, the sensitivity analyses were conducted to evaluate the robustness of the findings, and these analyses confirmed that models B1 and B2 still demonstrated excellent fit.

Our study has several other limitations. The main is the cross-sectional design and retrospective self-assessment of all analysed constructs on a relatively small sample. Future research should include a larger number of participants and a follow-up design with at least three time points to enable analyses of causal relationships using more advanced modelling approaches. Future research would also benefit from more reliable assessment of sleep, like standardised sleep quality scales, diaries, and actigraphy, which would improve the reliability of measurements and the analyses of responses given in a precise chronological order. This would also enable more precise causal modelling and interpretations. Furthermore, this study is limited to helping professionals, in whom pronounced effects and higher variability are expected. Future research should include other skilled professionals to be more representative for highly educated working population in general. Additional information on workrelated psychosocial factors other than working hours and shift work (e.g. work load, breaks and rest periods between shifts, level of control, leadership type, organisational climate, job satisfaction) could also prove useful for the analysis of mental health variables in a working population (53, 55).

To conclude, long working hours in highly educated workers in helping professions is a serious health and safety issue, both for the workers and for the recipients of their services. Our findings corroborate reports of the synergistic effect of daytime and nighttime functioning on individual’s mental health (21). Much of the relationship between working hours and anxiety can be explained via the effects of long working hours on shorter sleep duration on workdays, which negatively impacts perceived stress levels, which in turn contributes to higher levels of anxiety.
